# Bacteriological quality of household drinking water and cholera risk in the Greater Accra Region, Ghana

**DOI:** 10.11604/pamj.2025.50.39.45599

**Published:** 2025-01-31

**Authors:** Anthony Zunuo Dongdem, Bismark Sarfo, Adolphina Addo-Lartey, David Nana Adjei, Gifty Boateng, Wisdom Takramah, Maxwel Afetor, Grace Ababio, Gideon Kye-Duodu, Benjamin Kwasi Offei, Seth Owusu-Agyei, Francis Anto

**Affiliations:** 1 Department of Epidemiology and Biostatistics, Fred N. Binka School of Public Health, University of Health and Allied Health Sciences, Ho, Volta Region, Ghana,; 2 Department of Epidemiology and Disease Control, School of Public Health, College of Health Sciences, University of Ghana, Legon, Accra, Ghana,; 3 School of Biomedical and Allied Health Sciences, College of Health Sciences, University of Ghana, Korle-Bu, Accra, Ghana,; 4 National Public Health and Reference Laboratory, Ghana Health Service, Korle-Bu, Accra, Ghana,; 5 Information, Monitoring and Evaluation (IME) Department, Ghana Health Service, Ho Polyclinic, Ho, Ghana,; 6 Department of Biochemistry, University of Ghana Medical School, College of Health Sciences, University of Ghana, Korle-Bu, Accra, Ghana,; 7 Soil and Environmental Science Laboratory, BNARI, Ghana Atomic Energy, Accra, Ghana,; 8 Institute of Health Research, University of Health and Allied Health Sciences, Ho, Volta Region, Ghana

**Keywords:** Household water, contamination, coliform, cholera-risk, water source

## Abstract

**Introduction:**

the bacteriological quality of drinking water plays a critical role in preventing waterborne diseases. In Ghana, there is water scarcity and many communities depend on contaminated water sources for their domestic use. This study aimed to assess the bacteriological quality of household drinking water in both cholera endemic and non-endemic areas in Greater Accra Region.

**Methods:**

a community-based cross-sectional comparative survey in cholera endemic and non-endemic communities was conducted. A total of 480 drinking water samples were collected. The membrane filtration technique was used for the quantification of coliform counts and Vibrio counts. The bacteria were further identified and characterized. The Kruskal Wallis rank test was used to determine any significant variations in the means of the log-transformed bacteria counts among specific factor variables.

**Results:**

drinking water samples were contaminated with coliform counts exceeding the zero colony-forming units per 100 ml standard in most communities across cholera endemic and non-endemic areas. Vibrio counts were detected in all household water stored in vessels. Further characterization identified predominantly Klebsiella pneumonia and Escherichia coli. The coliform contamination levels were significantly higher in water stored in vessels compared to water directly obtained from the source. The contamination levels were generally higher during the wet season than the dry season.

**Conclusion:**

the household's stored drinking water and direct water sources were highly contaminated with coliform bacteria, posing a significant risk for the transmission of pathogenic waterborne diseases. Therefore, the need to implement an effective water treatment strategy to improve the quality of drinking water.

## Introduction

**Figure 1:**
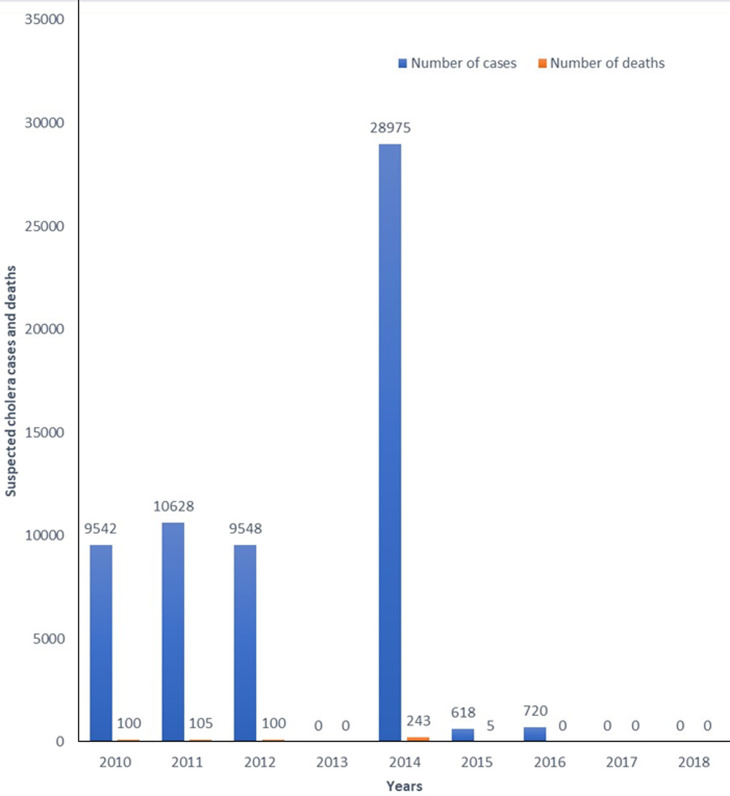
distribution of cholera cases and deaths in Ghana from 2010 to 2018

Water is a necessity for sustaining life and maintaining health. Access to safe, clean drinking water is a fundamental human right. However, billions of people globally, particularly in developing countries, are still deprived of this necessity [1,2]. Among those with access to water, millions drink from water sources contaminated with excreta [1], increasing the risk of waterborne diseases such as cholera, typhoid, and dysentery. In Ghana, waterborne diseases are a significant public health concern, with an estimated incidence of 25.6% [3]. Among these diseases, cholera is the most prevalent and consequential. From 2010 to 2018, Ghana reported a cumulative total of 60,031 cholera cases resulting in 553 deaths (Figure 1) [4-6]. The worst outbreak was in 2014 when over 28,000 cases and more than 240 deaths were recorded [7]. Although the cholera outbreaks frequently affected the coastal regions (i.e. Western, Central, Volta, and Greater Accra), the Greater Accra Region reported most of the cases each year [7,8]. Cholera is caused by *Vibrio cholerae*, an acute diarrheal disease that can lead to severe dehydration and even death if left untreated.

The disease is primarily transmitted through the consumption of contaminated water or food, with contaminated water being the most significant source of infection. Cholera outbreaks are known to occur in regions where sanitation and hygiene practices are suboptimal and access to safe drinking water is limited [9,10]. According to the WHO/UNICEF Joint Monitoring Programme (JMP) for Water, Sanitation, and Hygiene (WASH), only 44.5% of Ghana's population has access to safely managed drinking water and limited sanitation. Additionally, 17.2% of the population practices open defecation, while 41.7% have access to basic sanitation services - a situation of significant concern [11]. The Greater Accra Region, located in Southern Ghana, is a densely populated area with rapid urbanization and inadequate water and sanitation infrastructure. The region has experienced recurrent cholera outbreaks over the years, resulting in a significant number of deaths [7,12]. The lack of access to safe drinking water, coupled with poor sanitation practices, makes the population vulnerable to cholera infection. Understanding the bacteriological quality of household drinking water is crucial in assessing the risk of cholera transmission within communities. The bacteriological analysis provides insights into the presence and levels of indicator microorganisms, such as faecal coliforms and *Escherichia coli* (*E. coli*), which serve as markers for faecal contamination [13]. High levels of these indicator bacteria indicate the potential presence of pathogens, including *V. cholera* [13].

Several factors can contribute to the deterioration of drinking water quality, such as inadequate water treatment, contaminated sources, and improper storage and handling practices at the household level [14-16]. Identifying these factors and assessing their impact on water quality is essential for designing effective interventions to reduce the risk of cholera transmission and improve public health outcomes. This study aimed to investigate the bacteriological quality of household drinking water and its association with cholera risk in the Greater Accra Region. The presence and levels of faecal indicator bacteria were assessed in the household drinking water samples collected from different communities in the cholera endemic and non-endemic communities in the Greater Accra Region.

## Methods

**Figure 2:**
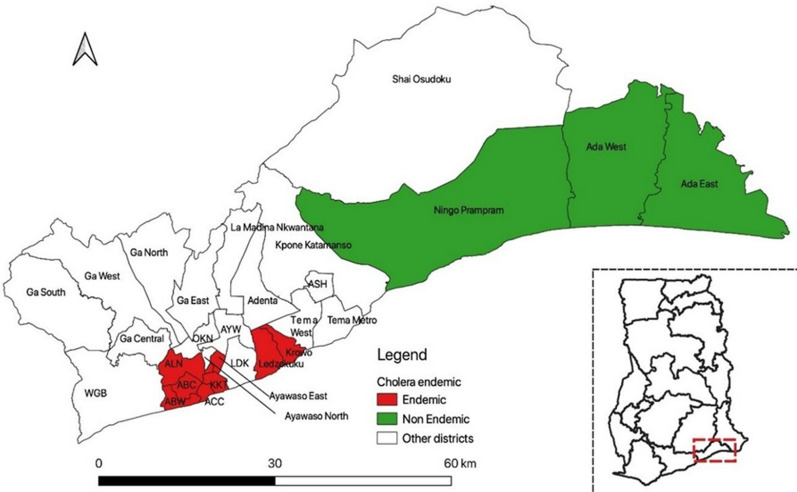
map of cholera endemic and non-endemic areas in the Greater Accra Region

**Description of the study area:** this study was carried out in the Greater Accra Region (GAR), one of the 16 administrative regions of Ghana. It is in the southern part of the country and bordered by the Central Region to the west, the Volta Region to the east, the Eastern Region to the north, and the Gulf of Guinea to the south (Figure 2). It has the smallest land area of the 16 regions, with an estimated population of 5,055,805 [17]. The capital city of Ghana, Accra is also located in the region with a daily influx of people from within and outside the country. The region is the most urbanized and has the highest population density in the country [18]. There are highly populated slums, squatters, and informal settlements in parts of the region due to the rapid urbanization being experienced [19,20]. The region has 29 districts including two metropolitan areas (i.e. Accra and Tema) and 23 municipalities. Twelve communities within the Accra Metropolitan Assembly, such as Agbogbloshie, James Town, Adabraka, Kaneshie, Maamobi, Nima, Chorkor, Mamprobi, Agege, and Dansoman as well as Teshie and Nungua in the Ledzokuku Krowor Municipality [7,21]. In contrast, there are other communities in the region classified as cholera non-endemic including Dawhenya North, Kofikope, Awhiam, and Dawa in the Ningo Prampram district; Addokope, Dogobom, Anyaman East, Adjumanikope in the Ada West district; Adedetsekope, Angomya-Ada, Kophemm Kasseh and Asigbekope in the Ada East district, as shown in Figure 2 [7,21].

**Study design:** a community-based cross-sectional comparative design was used for this study. The study area was classified into cholera endemic and non-endemic communities. The cholera endemic communities were communities in the Accra Metropolitan Area, Ledzokuku, and Krowor Municipal District which have consistently reported cases of cholera over the past five years (2011 to 2015), preceding the start of this study, with evidence of local transmission [7,21]. The cholera non-endemic communities were communities in the Ningo Prampram, Ada West, and East Districts that have rarely recorded any case of cholera over the same period with no evidence of local transmission in the area [7,21]. Households were randomly selected and their drinking water was sampled for bacteriological water quality assessment. The study was conducted in the wet (April to August 2019) and dry (November 2019 to January 2020) seasons.

**Sample size determination:** the sample size was determined using the Cochran´s formula:


$$N=\frac{z^{2}p\left(1-p\right)}{e^{2}}$$

$$N=\frac{{\left(1.96\right)}^{2}0.838\left(1-0.838\right)}{{\left(0.05\right)}^{2}}=208.608$$

By assuming z: 95% confidence interval; e: 5% margin of error; and p: proportion of households drinking water contaminated with *V. cholerae* as 83.8% reported in Accra Metropolis [22], to obtain a minimum sample size of 209 for both cholera-endemic and non-endemic communities for the dry season and doubled to include the wet season.

**Sampling technique:** a multistage cluster sampling approach was used to determine which household should be sampled. The first cluster represented a cholera-endemic or non-endemic community. The second stage involved the random selection of 12 communities from both the endemic and also, non-endemic communities. The households were subsequently listed for each community and 9 households were randomly selected by a ballot. An additional water sample was collected from the primary drinking water source in each of the communities (e.g. public tap, pipe, or dam) to serve as a control sample. The 12 selected cholera-endemic communities were; Agbogbloshie, James Town, Adabraka, Dansoman, Agege, Mamprobi, Chorkor, Kaneshie, Nima, Maamobi, Teshie, and Nungua. Whereas the 12 non-endemic communities were; Addokope, Dogobom, Adjumanikope, Anyaman, Adedetsekope, Agomya-Ada, Kopehem-Kasseh, Asigbeykope, Awhiam, Dawa, Kofikope, and Dawhenya North. In total, 216 water samples were collected from household storage vessels and 24 from the direct water sources were collected in cholera-endemic communities during the wet and dry seasons to assess for temporal variation in water quality. The same number of samples were also collected in the cholera non-endemic communities.

**Water sample collection:** drinking water samples from households were collected to assess bacteriological quality. Heads of households or representatives were asked to provide a cup of drinking water, as they would for a child or guest, to ensure the best available water quality was sampled. Approximately 500 ml of stored household drinking water was collected using the container usually used to scoop water from the storage container. The water was transferred into a sterile plastic bottle. For households that consumed sachet water, the edge of the sachet was decontaminated by cleaning with 70% ethanol, cut aseptically with sterile scissors, and the water was poured into the sterile plastic bottle. If bottled water was consumed, they were collected and labelled. Water samples from source points (e.g. pipes, standpipes, or unprotected wells) were collected directly. For tap or pipe water, the tap exterior was cleaned with 70% ethanol and the water was allowed to flow at maximum for about one minute before collection into the sterile plastic bottle [23]. To neutralize residual chlorine in pipe-borne water, 0.5 ml of 10% sodium thiosulphate was added to the pipe-borne water before transporting it to the laboratory [23]. The water samples collected from the field were transported in insulated boxes at 4°C to the laboratory within six hours. In case of a delay, the water samples were stored in the refrigerator (2°C to 8°C) and processed within 24 hours.

**Ethical Statement:** prior to the initiation of this study, ethical approval was given from the Ghana Health Service Ethical Review Committee (GHS-ERC) under the approval number GHS-ERC 006/01/19. Community leaders granted initial permission, followed by written consent from the heads of households or representatives, using a structured consent form. The informed consent form was explained to the participants in a dialect they could understand by a research assistant. The participants were provided with information regarding the study's purpose, rationale, selection process, procedures, and interview duration. Those individuals who willingly agreed to take part in the study expressed their consent by either signing the consent forms or providing a thumbprint.

**Laboratory analysis:** the water samples were analyzed in accordance with the American Public Health Association (APHA) standard methods for examining drinking and wastewater using the membrane filtration method as described by Eaton, Clesceri [24]. In instances where the growth was heavy and uncountable (i.e. greater than 200 colony count), 10 ml, 1 ml, 0.1 ml, or 0.01 ml of the water was used and diluted with buffered peptone water to make up the 100 ml before filtration. The 0.45µm membrane filter was aseptically removed and placed in the respective appropriate media. The inoculated Plate Count Agar (PC) and Violet Red Bile (VRB) agar were incubated at 36°C ± 1, while the Eosin Methylene Blue (EMB) agar and Thiosulfate-Citrate-Bile-Sucrose (TCBS) agar were incubated at 44°C ± 1. The plates were observed after 24 hours and 48 hours. The plates for PC and VRB were counted and estimated respectively for the total viable count and total coliform. The EMB was estimated for faecal coliforms (thermotolerant coliforms) and TCBS for suspected *Vibrio* species.

**Sub-culturing of colonies:** the morphological characteristics of the colonies on the filter membrane were described. Colonies that appeared as; large, smooth, yellow, slightly flattened, and translucent peripheries on the TCBS medium were presumptively identified as *Vibrio* species. These colonies were sub-cultured onto another TCBS plate for purification. The suspected *Vibrio* colonies were further sub-cultured on Tryptone Soy Agar (TSA) (a non-selective media) for biochemical testing and serotyping. Other suspected coliform bacteria growing on the PC agar, VRB, and EMB were sub-cultured on the MacConkey agar plate for purification. Distinct colonies were sub-cultured on TSA for presumptive identification and biochemical testing using the Mini Antigenic Profile Index 20 Enterobacteriaceae (API 20E) (bioMérieux, France) for confirmation.

**Quality control:** all the containers used for the collection of water samples from the field were sterilized by autoclaving and checking with an autoclave tape to ensure sterilization. All the bacteriological media were prepared in accordance with the manufacturer´s instructions and dispensed into disposable pre-sterilized plastic petri dishes. A sterility test was performed on each batch of media prepared by incubating a plate at 35 to 37°C overnight (18 to 24 hours) and examined for any contamination. A media performance test was also conducted by culturing in-house or reference bacteria strains obtained from the National Public Health and Reference Laboratory, Ghana to assess the growth characteristics of the media. The reference strains and in-house control strains were used as reference points to validate the results of the biochemical tests. Blank membrane filtration was carried out after every fifth sample. This was to ensure growths on the filter membrane were not influenced by the laboratory conditions. The filtration techniques were carried out in a laminar flow hood to avoid contamination as much as possible.

**Data processing and analysis:** result of the analysis of the water samples were entered into Microsoft Excel. It was exported into STATA software version 14.0 (State Cooperation, USA), cleaned and analyzed. Descriptive statistics such as means and standard deviation for continuous variables and frequencies and percentages for categorical variables were computed. Inferential statistics were applied to estimate the water parameters such as the total coliform, faecal coliform and vibrio counts. Kruskal Wallis rank test, a non-parametric equivalent of the Analysis of Variance (ANOVA) test was performed to determine whether there were any significant differences between the means of the log of bacteria counts among some selected factor variables.

## Results

**Assessment of the bacteriological quality of drinking water and cholera risk in Greater Accra Region:** a total of 480 household drinking water samples were collected and analyzed, with 240 samples from cholera-endemic communities and 240 from non-endemic communities. Samples were collected during the wet and dry seasons, with 120 samples investigated per season. The water was tested for *Vibrio cholerae* and indicator bacteria including coliforms.

**Cholera-endemic communities:** in the endemic communities, 24 water samples were collected directly from private (4) and public (20) pipes water sources in the dry and wet seasons. High levels of total coliform counts (TCC) were observed in ten communities with a mean count of 7.73 log cfu/100ml and 9.86 log cfu/100ml respectively in the dry and wet seasons as shown in Table 1. Feacal coliforms (FCC) were generally present in the private pipe water sources in both seasons except in a few communities: Agege and Dansoman which did not record any counts in both season. *Vibrio* counts (VC) were absent in the private pipe water sources except in Mamprobi which recorded 6.91 log cfu/100 ml in the wet season. The direct public pipe also had a high mean TCC of 7.82 log cfu/100 ml in the dry season and 9.64 log cfu/100 ml in the wet season. The mean FCC was also found to be 5.24 log cfu/100 ml and 10.06 log cfu/100 ml respectively in the dry and wet seasons. No VC was observed in the communities (Agbogbloshie and James Town) during both seasons. The differences in the mean rank scores for the log of TCC (χ^2^= 0.182, p = 0.669), FCC (χ^2^= 2.591, p = 0.107), and VC (χ^2^= 0.200, p = 0.655) were assessed among the direct water sources, with no statistically significant differences observed, as shown in Table 2.

**Table 1:** mean log of bacterial counts in the dry and wet seasons across some selected background characteristics in endemic communities

Study variable	Characteristics	TCC (log cfu/ 100 ml)		FCC (log cfu /100 ml)		VC (log cfu /100 ml)	
		Dry	Wet	Dry	Wet	Dry	Wet
Direct water sources	Community	Mean (sd)	Mean (sd)	Mean (sd)	Mean (sd)	Mean (sd)	Mean (sd)
Private pipe	Adabraka	8.16	10.04	5.35	9.30	0	0
	Nungua	7.24	12.10	4.50	10.57	0	0
	Agege	8.07	8.85	0	0	0	0
	Chorkor	7.60	8.52	4.60	0	0	0
	Dansoman	7.74	9.39	0	0	0	0
	Kanehsie	8.16	10.93	0	6.91	0	0
	Mamobi	8.04	10.46	4.79	8.29	0	0
	Mamprobi	6.91	9.61	5.13	0	0	6.91
	Nima	7.60	8.52	4.79	8.52	0	0
	Teshie	7.74	10.20	4.50	10.13	0	0
	Total	7.73(0.41)	9.86(0.36)	4.81(0.32)	8.95(1.34)	0	6.91
Public pipe	Agbogbloshie	8.04	10.60	5.13	10.34	0	0
	James town	7.60	10.78	4.79	9.80	0	0
	Total	7.82(0.31)	9.64(1.29)	5.24(0.65)	10.06(0.38)	0	0
Water stored in vessels	Private pipe	8.26(0.38)	10.76(1.13)	5.53(0.45)	10.04(1.09)	2.40 (0.69)	9.91(1.15)
	Public pipe	8.49(0.37)	11.75(0.38)	5.80(0.44)	11.16(0.76)	1.77(0.88)	9.76(1.52)
	Sachet	8.45(0.39)	10.51(1.08)	5.60(0.41)	10.18(0.99)	2.29(0.71)	10.49(0.57)

TCC: total coliform counts; FCC: feacal coliforms; VC: vibrio counts

**Table 2:** assessing the differences in log bacterial count among household drinking water stored in vessels and from direct water sources in endemic communities

	n	Rank sum	X^2^ with ties	P-value
**Total coliform count**				
Direct source			0.182	0.669
Private pipe	4	55.50		
Public pipe	20	244.50		
Water stored in vessels			7.704	0.021
Private pipe stored	110	12068.50		
Public pipe stored	23	3198.50		
Sachet water	83	8169		
**Faecal coliform count**				
Direct source			2.591	0.107
Private pipe	4	70.50		
Public pipe	20	229.50		
Water stored in vessels			17.156	<0.001
Private pipe stored	110	12170		
Public pipe stored	23	3532.50		
Sachet water	83	7733.50		
**Vibrio count**				
Direct source			0.200	0.655
Private pipe	4	48		
Public pipe	20	252		
Water stored in vessels			12.101	0.002
Private pipe stored	110	12094		
Public pipe stored	23	3289		
Sachet water	83	8053		

In the endemic communities, stored household drinking water was also analyzed in both seasons. Of the 216 water samples; 110 were private pipes, 23 were public pipes, and 83 were sachet water. The highest mean TCC and FCC in both seasons were found in stored public pipe water compared to stored private pipe water and sachet water in the households (Table 1). Suspected vibrio counts were present in all household-stored drinking water samples. However, when the differences between the bacterial mean counts were assessed for the stored household drinking water, the mean rank score of the log of TCC (χ^2^= 7.704, p = 0.021), FCC (χ^2^= 2.591, p<0.001) and VC (χ^2^= 12.101, p = 0.002) differed significantly (Table 2). The bacterial counts were generally higher in the wet season than the dry season during the period for both the water collected directly from the source and that stored in vessels in the households.

**Cholera non-endemic communities:** in the non-endemic communities also, 24 direct water sources were sampled from private pipes (4), public pipes (16), and unprotected wells (4) in both seasons (Table 3). The direct private piped water sources were found in Dawhenya and Kofikope and recorded a mean TCC of 8.57 log cfu/100 ml in the dry season and 10.12 log cfu/100 ml in the wet season. The mean FCC was 4.50 log cfu/100 ml and 8.61 log cfu/ml in the dry and wet seasons respectively, with no recorded VC. The public pipe samples recorded a mean FCC of 8.44 log cfu/ml in the dry season and 9.64 log cfu/ml in the wet season. The mean FCC was 5.24 log cfu/100 ml in the dry season and 9.39 log cfu/100 ml in the wet season, although six of the communities (Dawa, Dogobom, Adedetsekope, Asigbeykope, Awhiam, Adjumanikope) did not record any feacal coliforms. Anyaman East community, on the other hand, recorded the highest TCC and FCC in both seasons. Except for Awhiam community which had a VC of 6.91 log cfu/100 ml, all other communities did not record any counts. The unprotected wells also recorded a mean TCC of 8.61 log cfu/100 ml in the dry season and 10.20 log cfu/100 ml in the wet season. The mean FCC was 5.53 log cfu/100 ml in the dry season and 9.45 log cfu/100 ml in the wet season with a mean VC of 10.31 log cfu/100 ml.

**Table 3:** mean log of bacterial counts in the dry and wet seasons across some selected background characteristics in non-endemic communities

Study variable	Characteristics	TCC (log cfu/ 100 ml)		FCC (log cfu /100 ml)		VC (log cfu /100 ml)	
		Dry	Wet	Dry	Wet	Dry	Wet
**Direct water sources**	**Community**	**Mean(sd)**	**Mean(sd)**	**Mean(sd)**	**Mean(sd)**	**Mean(sd)**	**Mean(sd)**
Private pipe	Dawhenya	8.48	10.09	0	8.70	0	0
	Kofikope	8.66	10.16	4.50	8.52	0	0
	Total	8.57(0.13)	10.12(0.06)	4.50	8.61(0.13)	0	0
Public pipe	Addokope	8.63	9.21	4.79	0	0	0
	Dawa	8.29	0	0	0	0	0
	Dogobom	8.34	8.00	0	0	0	0
	Adedetsekope	8.27	0	0	0	0	0
	Anyaman East	8.81	10.60	5.70	9.39	0	0
	Asigbeykope	8.57	0	0	0	0	0
	Awhiam	8.48	10.76	0	0	0	6.91
	Adjumanikope	8.16	0	0	0	0	0
	Total	8.44(0.22)	9.64(1.29)	5.24(0.65)	9.39	0	6.91
Unprotected dug well	Angomya-Ada	8.41	11.00	5.67	9.80	0	10.31
	Kopeheum Kasseh	8.81	9.39	5.39	9.10	0	0
	Total	8.61(0.28)	10.20(1.14)	5.53(0.19)	9.45(0.49)	0	10.31
Water stored in vessels	Private pipe	8.92(0.26)	11.10(0.68)	6.16(0.36)	10.27(0.71)	1.84(0.62)	9.92(1.24)
	Public pipe	8.77(0.26)	10.45(1.13)	6.03(0.34)	9.59(1.08)	2.23(0.92)	9.73(1.08)
	Sachet	8.88	12.21	5.74	10.93	0	0
	Unprotected dug well	8.81(0.31)	11.16(0.61)	6.10(0.40)	10.39(0.77)	2.48(0.59)	11.19(0.78)
	Dam	9.05(0.23)	11.83(0.13)	6.44(0.23)	10.60(0.18)	2.91(0.77)	9.24(0.34)

TCC: total coliform counts; FCC: feacal coliforms; VC: vibrio counts

The differences in the mean rank score between the log of FCC among the direct water sources were assessed and found to be statistically significant (χ^2^= 10.212, p = 0.006). Whereas, the mean rank score of the log of TCC and VC among the direct water sources did not differ (χ^2^= 5.171, p = 0.075: χ^2^= 2.029, p = 0.363) as shown in Table 4. The two hundred and sixteen (216) households stored drinking water in the non-endemic communities made up of; 34 private piped water, 136 public pipe water, 36 unprotected dug wells, 8 dam water, and 2 sachet water stored in the household. Except for VC which was not found in sachet water, TCC, FCC, and VC were generally present in all household water stored in vessels for the period. The bacterial counts in the stored water samples were higher than those collected from the direct water sources. The TCC and FCC were generally observed to be higher during the wet season than the dry season. The differences between the bacterial mean counts were assessed for the household drinking water stored and a statistically significant difference was found in the mean rank score of the log of TCC and FCC (χ^2^= 14.165, p = 0.007: χ^2^= 33.901, p<0.001), while the mean rank scores for log of VC (χ^2^= 6.051, p = 0.195) was not significant (Table 4).

**Table 4:** assessing the differences in log bacterial count among household drinking water stored in vessels and from direct water sources in non-endemic communities

	n	Rank sum	X^2^ with ties	P-value
**Total coliform count**				
Direct source			5.171	0.075
Private pipe	4	67.50		
Public pipe	16	163		
Unprotected dug well	4	69.50		
Water stored in vessels			14.165	0.007
Unprotected dug well	36	4423.0		
Dam water	8	1189.5		
Private pipe	34	4357.5		
Public pipe	136	13168.0		
Sachet	2	298.0		
**Faecal coliform count**				
Direct source			10.212	0.006
Private pipe	4	63.50		
Public pipe	16	155.50		
Unprotected dug well	4	81		
Water stored in vessels			33.901	<0.001
Unprotected dug well	36	4972.0		
Dam	8	1257.5		
Private pipe	34	4743.0		
Public pipe	136	12205.5		
Sachet	2	258.0		
**Vibrio count**				
Direct source			2.029	0.363
Private pipe	4	46		
Public pipe	16	195.50		
Unprotected dug well	4	58.50		
Water stored in vessels			6.051	0.195
Unprotected dug well	36	4206.5		
Dam	8	1152.0		
Private pipe	34	3525.5		
Public pipe	135	14226.0		
Sachet	2	110.0		

**Figure 3:**
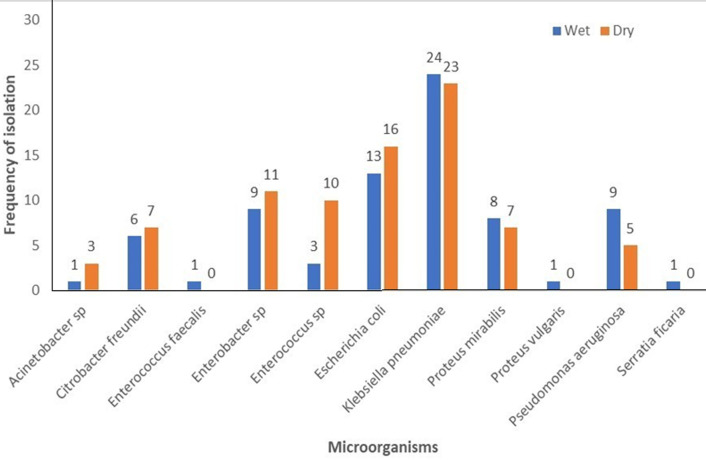
frequency of isolation of microorganisms in household drinking water during the wet and dry seasons in the cholera endemic communities

**Bacterial isolates and cholera risk:** in the cholera-endemic communities, 120 household water samples were collected per season. Positive bacterial growth was found in 76 (63.3%) of samples in the wet season and 82 (68.3%) in the dry season, with *Klebsiella pneumonia* 24 (31.6%) and *Escherichia coli* 13 (17.1%) as the predominant bacteria. No *Vibrio cholerae s*trains were isolated (Figure 3).

**Figure 4:**
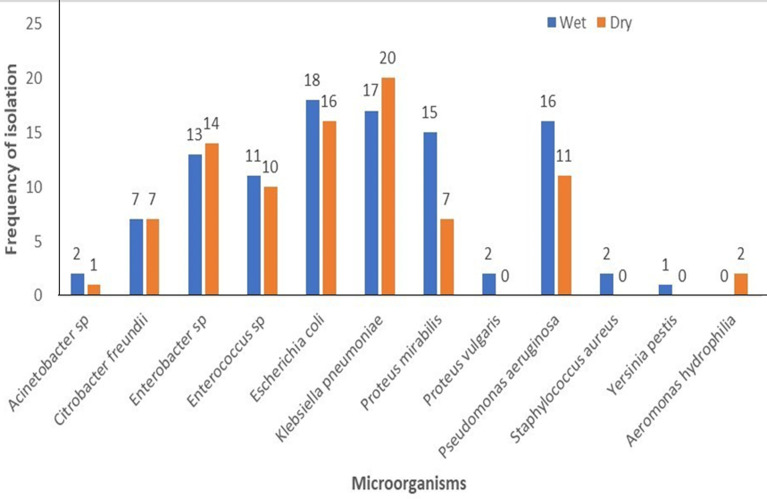
frequency of isolation of microorganisms in household drinking water during the wet and dry seasons in the cholera non-endemic communities

**Figure 5:**
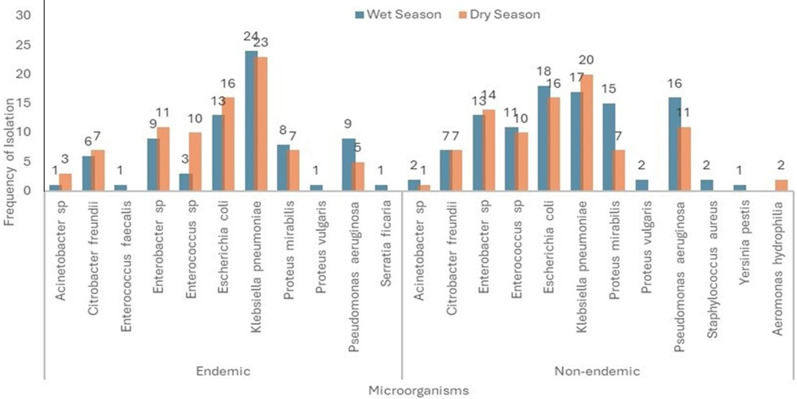
seasonal comparison of microorganism isolation frequency in cholera endemic and non-endemic communities

In the non-endemic communities, 104 (86.7%) of water samples in the wet season and 18 (73.3%) in the dry season showed bacterial growth. *Escherichia coli* 18 (17.3%) and *Klebsiella pneumonia* 17 (16.4%) were most common in the wet season, while *Klebsiella pneumonia* 20 (22.7%) and *Escherichia coli* 16 (18.2%) were most frequent in the dry season. Multiple bacterial species were detected in some samples, but no *Vibrio cholerae* was isolated (Figure 4). The overall frequency of bacterial isolation was higher in the cholera-endemic communities compared to non-endemic communities. Certain bacterial species were found exclusively in endemic communities, such as *Enterococcus faecalis* and *Serratia ficaria*. Conversely, *Staphylococcus aureus, Yersinia pestis*, and *Aeromonas hydrophilia* were isolated only in non-endemic communities (Figure 5).

## Discussion

The bacteriological quality of water can be affected by the natural presence of microorganisms or contamination from human activities, increasing the risk of cholera and other diarrhoeal diseases [13]. In this study, we assessed the bacteriological quality of both direct water sources and stored household drinking water. In the cholera-endemic communities, drinking water was typically sourced from private pipes or public pipes. In the non-endemic communities, however, water sources included private pipes, public pipes and unprotected wells. This study revealed that water samples collected from direct pipes, public pipes, and unprotected wells during the dry and wet seasons were frequently contaminated. These samples showed total and/or faecal coliform counts exceeded the WHO and Ghana standards of zero (0) cfu/100 ml for drinking water [25,26]. This contamination was observed in both endemic and non-endemic communities, making them unwholesome for consumption. These findings are consistent with other studies that recorded high total and faecal coliform counts in directly collected piped and well water [27,28]. The high bacterial counts in the piped water sources may be attributed to the laying of pipelines in gutters and the inadequate maintenance of the pipelines. Additionally, insufficient chlorination may likely contribute to the high bacterial counts. This aligns with the findings of Karikari *et al*. [29] in an earlier study that reported low chlorine levels and the presence of faecal coliform in the Accra water network. The comparison of bacterial counts between the direct piped and public taps in the endemic communities showed no statistical significance, suggesting similar levels of contamination in both sources. Bacteria counts in the non-endemic communities were similar across the different water sources, with the exception of faecal coliform counts, which showed significant variations between private, public and unprotected well water.

The private and public piped water, supplied by the Ghana Water Company, may likely reflect their quality-of-service delivery that needs improvement to reduce the risk of cholera and other diarrhoeal diseases. The high faecal coliform counts observed in unprotected wells may be due to run-off water contamination, particularly during the rainy season. Despite this, the direct main public tap water in Dawa, Aedetsekope, Asigbeykope, and Adjumanikope did not record any coliform counts during the wet season and met the quality standards. These communities have a community public standpipe centrally located at a point in the community and connected to either a borehole or directly to an elevated storage tank, resulting in fewer pipeline connections, unlike the endemic communities where the public standpipes are connected to the general pipeline distribution network and more susceptible to contamination. In terms of seasonality, both total and faecal coliform counts were higher during the wet season compared to the dry season in both the endemic and non-endemic communities. This finding agrees with other previous studies which also reported elevated total and faecal coliform counts in the wet season [30,31]. The increase may be attributed to the environmental conditions surrounding some water sources in these communities which likely deteriorate further during periods of heavy rainfall.

In this study, the water stored for drinking by the households was also assessed for the level of contamination. This source water was collected and stored mostly in plastic containers and a few in earthenware/pots. The study results revealed that irrespective of the water source (a private piped, public piped, sachet water or well) the contamination levels for total and faecal coliforms were higher than that directly collected from the water source. In the endemic communities, our study found a significant difference between the bacterial contamination levels and the water sources stored in the household. This was similarly observed in the non-endemic communities, except the *Vibrio* count which did not show any significant differences with the water sources stored. This study is consistent with the findings of Meierhofer *et al*. [32] in Kenya which found higher contamination levels in the storage containers. The reports of Boateng *et al*. [33] and Agensi *et al*. [34] in Tamale, Ghana and Kisoro, Uganda respectively, similarly align with this study's findings. The cleanliness of the storage vessels, methods of drawing water from the storage container and the length of the storage were implicated in the high bacterial counts [15,16]. The study findings revealed that sachet water had similarly high levels of contamination with total and faecal coliforms as the private pipe and public tap water. Earlier studies in Ghana and Nigeria have demonstrated that water can be contaminated with faecal coliform and other pathogenic bacteria, making it unwholesome for consumption [35-37]. Semey *et al*. [38] suggested that such contamination may result from unhygienic handling by factory workers during production or from the use of contaminated water sources that undergo no further treatment before. Additionally, Dzodzomenyo *et al*. [39] noted that some sachet water companies operate without formal registration by the Food and Drugs Authority, potentially violating the quality standards for quality water production. Aslan *et al*. [35] further highlighted issues such as sachet bags lacking batch numbers, manufacturing dates or treatment information. This is worrisome as the reliance on sachet water may not provide protection against waterborne diseases like cholera.

Further analysis was conducted to confirm the suspected coliform and *Vibrio* isolates that were mostly detected in the household drinking water storage in both seasons. The most frequently isolated organisms in both seasons were *Escherichia coli* and *Klebsiella pneumoniae*. The presence of these organisms indicates the possibility of the presence of potentially harmful bacteria and a high risk of their transmission. The suspected *Vibrio* counts observed were confirmed to be negative for *Vibrio cholerae*, instead were identified as *Pseudomonas aeruginosa, Proteus sp*. and *Aeromonas hydrophilia*. Our findings did not detect the presence of *V. cholerae* and contrasts that of Yirenya-Tawiah *et al*. [22] who found *Vibrio cholerae* O1 strains in household drinking water storage in some communities in Accra just before the major cholera outbreak in 2014. The absence of *V. cholerae* in the current study could explain the reason for the absence of a cholera outbreak during the study period. The findings further strengthen the importance of regular environmental surveillance to predict cholera outbreaks.

**Strength and limitation:** the findings of this study indeed have significant implications for understanding household drinking water quality and its relationship with cholera transmission in the Greater Accra Region. By examining the current state of water quality, the study provides valuable insights that can inform policymakers, public health authorities, and other stakeholders in their efforts to combat cholera and improve overall water and sanitation practices in the region. However, the type of storage vessels used was not accounted for and could be a significant limitation. Different types of storage vessels may have varied levels of susceptibility to contamination, and this could impact the overall quality of the water. To address these limitations, future research or follow-up studies could consider incorporating the assessment of storage vessel types and their impact on water quality.

## Conclusion

The household drinking and direct water sources were contaminated with total and faecal coliform counts that exceeded the Ghana and WHO recommendations in both the endemic and non-endemic communities. The bacterial counts followed a seasonal pattern peaking during the wet season than the dry season, possibly resulting from contamination of water sources from run-off flood waters. *Escherichia coli* and *Klebsiella pneumonia* were the most common coliforms recovered with no *V. cholerae* strains isolated from both the direct or stored household drinking water. The presence of these coliform bacteria gives an indication of faecal contamination and a high risk of waterborne diseases. It is important, therefore, to educate household members to treat their drinking water at the point of use to avoid drinking contaminated water and reduce the risk of transmission of cholera and other diarrhoeal pathogens. It is also important for the Ghana Standard Authority to regularly monitor the bacteriological quality of the sachet water manufacturing companies to ensure their operations conform to the quality standards since it is the most preferred source of drinking water.

### What is known about this topic

- It is well recognized that the bacteriological quality of drinking water is influenced by naturally occurring microbes and human activity;
- Water from private and public pipes, as well as unprotected wells, often contains coliform bacteria making them unsafe for consumption;
- Water stored in households frequently has higher bacterial contamination levels compared to water directly collected from the source.

### What this study adds

- The study confirms that both cholera-endemic and non-endemic communities were exposed to faecal contamination of their drinking water and were at risk of cholera and other diarrhoea disease outbreaks;
- The study provides evidence of Escherichia coli and Klebsiella pneumonia in household drinking water storage, indicating faecal contamination, although Vibrio cholerae strains were not detected, ongoing monitoring is essential to predict outbreaks;
- This study adds evidence that sachet water, which households often consider safer, can also be significantly contaminated; this contamination may result from poor handling practices and failure to comply with production standards.

## References

[1] Bain R, Cronk R, Hossain R, Bonjour S, Onda K, Wright J (2014). Global assessment of exposure to faecal contamination through drinking water based on a systematic review. Trop Med Int Health.

[2] Wright J, Gundry S, Conroy R (2004). Household drinking water in developing countries: a systematic review of microbiological contamination between source and point-of-use. Trop Med Int Health.

[3] Honlah E, Yao Segbefia A, Odame Appiah D, Mensah M, Atakora PO (2019). Effects of water hyacinth invasion on the health of the communities, and the education of children along River Tano and Abby-Tano Lagoon in Ghana. Cogent Social Sciences.

[4] Deen J, Mengel MA, Clemens JD (2020). Epidemiology of cholera. Vaccine.

[5] Donkor AG, Namaitijiang M (2019). The Cholera outbreak in Ghana. ESTÜDAM Halk Sagligi Dergisi.

[6] Basiru I, Arkorful VE, Ashu HA, Lukman S (2019). Challenges affecting sustainability of National Sanitation Day (NSD) programme in Ghana. Journal of Health and Medical Sciences.

[7] Ghana Disease Surveillance Department (2015). Cholera Hotspots in Greater Accra Region. Ghana Health Service.

[8] Apenteng JA, Korsah S, Tagoe M, Nortey NN, Korsah J, Wobetsey BD (2023). Outbreak of Cholera in Ghana: A Systematic Review from 2010 to 2020. Asian Journal of Research in Infectious Diseases.

[9] D'Mello-Guyett L, Gallandat K, Van den Bergh R, Taylor D, Bulit G, Legros D (2020). Prevention and control of cholera with household and community water, sanitation and hygiene (WASH) interventions: A scoping review of current international guidelines. PLoS One.

[10] Cronin AA, Shrestha D, Cornier N, Abdalla F, Ezard N, Aramburu C (2008). A review of water and sanitation provision in refugee camps in association with selected health and nutrition indicators-the need for integrated service provision. J Water Health.

[11] WHO/UNICEF (2022). Joint Monitoring Programme (JMP).

[12] Mengel MA, Delrieu I, Heyerdahl L, Gessner BD (2014). Cholera outbreaks in Africa. Curr Top Microbiol Immunol.

[13] World Health Organization (2017). Guidelines for drinking-water quality: first addendum to the fourth edition.

[14] Yeboah SI, Antwi-Agyei P, Domfeh MK (2022). Drinking water quality and health risk assessment of intake and point-of-use water sources in Tano North Municipality, Ghana. Journal of Water, Sanitation and Hygiene for Development.

[15] Lartey KA (2019). Assessing the Quality of Household Drinking Water in Selected Communities in the Akuapem South District.

[16] Asfaw HS, Reta MA, Yimer FG (2016). High enteric bacterial contamination of drinking water in Jigjiga city, Eastern Ethiopia. Ethiopian Journal of Health Development.

[17] Ghana Statistical Service (2014). 2010 Population and Housing Census District Analytical Report. Accra Metropolitan.

[18] Ghana Statistical Service (2013). 2010 Population and Housing Census. National Analytical Report.

[19] Amoako C (2016). Brutal presence or convenient absence: The role of the state in the politics of flooding in informal Accra, Ghana. Geoforum.

[20] Accra Metropolitan Assembly. United Nations Habitat (2011). Participatroy Slum upgrading and revention: Millennium city of Accra, Ghana. UN Habitat Annual report, Accra.

[21] Ghana Disease Surveillance Department (2016). 2014 to 2015 Cholera outbreak over report. Ghana. Ghana Health Service/Ministry of Health: Departmental Report.

[22] Yirenya-Tawiah DR, Darkwa A, Dzodzomenyo M (2018). Environmental surveillance for Vibrio cholerae in selected households´ water storage systems in Accra Metropolitan Area (AMA) prior to the 2014 cholera outbreak in Accra, Ghana. Environ Sci Pollut Res Int.

[23] Monica C (2006). District Laboratory Practice in Tropical Countries.

[24] Eaton AD, Clesceri LS, Rice EW, Greenberg AE (2005). APHA: standard methods for the examination of water and wastewater. American Public Health Association.

[25] World Health Organization (1993). Guidelines for drinking-water quality, 2nd edition Volume 1 - Recommendations.

[26] Ghana Standard Authority (2009). Limits for drinking water (GS 175-1).

[27] Tekpor M, Akrong MO, Asmah MH, Banu RA, Ansa ED (2017). Bacteriological quality of drinking water in the Atebubu-Amantin district of the Brong-Ahafo region of Ghana. Applied Water Science.

[28] Oyedum UM, Adabara NU, Kuta FA (2016). Comparative study of coliform contamination of public boreholes and pipe borne water systems in Bosso town, North Central, Nigeria. Journal of Applied Sciences and Environmental Management.

[29] Karikari AY, Ampofo JA (2013). Chlorine treatment effectiveness and physico-chemical and bacteriological characteristics of treated water supplies in distribution networks of Accra-Tema Metropolis, Ghana. Applied Water Science.

[30] Dongzagla A, Jewitt S, O'Hara S (2021). Seasonality in faecal contamination of drinking water sources in the Jirapa and Kassena-Nankana Municipalities of Ghana. Sci Total Environ.

[31] Odonkor ST, Mahami T (2020). Escherichia coli as a Tool for Disease Risk Assessment of Drinking Water Sources. Int J Microbiol.

[32] Meierhofer R, Wietlisbach B, Matiko C (2019). Influence of container cleanliness, container disinfection with chlorine, and container handling on recontamination of water collected from a water kiosk in a Kenyan slum. J Water Health.

[33] Boateng D, Tia-Adjei M, Adams EA (2013). Determinants of household water quality in the Tamale Metropolis, Ghana. Journal of Environment and Earth Science.

[34] Agensi A, Tibyangye J, Tamale A, Agwu E, Amongi C (2019). Contamination Potentials of Household Water Handling and Storage Practices in Kirundo Subcounty, Kisoro District, Uganda. J Environ Public Health.

[35] Aslan A, Rochani H, Oyibo O, Dotherow JE, Anderson KW, Beslin C (2020). Sources of microbiological contamination in sachet water from Ghana. Journal of Water, Sanitation and Hygiene for Development.

[36] Mosi L, Adadey SM, Sowah SA, Yeboah C (2019). Microbiological assessment of sachet water “pure water” from five regions in Ghana. AAS Open Res.

[37] Oluwafemi F, Oluwole ME (2012). Microbiological examination of sachet water due to a cholera outbreak in Ibadan. Nigeria.

[38] Semey MD, Dotse-Gborgbortsi W, Dzodzomenyo M, Wright J (2020). Characteristics of packaged water production facilities in Greater Accra, Ghana: implications for water safety and associated environmental impacts. Journal of Water, Sanitation and Hygiene for Development.

[39] Dzodzomenyo M, Fink G, Dotse-Gborgbortsi W, Wardrop N, Aryeetey G, Coleman N (2018). Sachet water quality and product registration: a cross-sectional study in Accra, Ghana. J Water Health.

